# Maternal Urinary Bisphenol A during Pregnancy and Maternal and Neonatal Thyroid Function in the CHAMACOS Study

**DOI:** 10.1289/ehp.1205092

**Published:** 2012-10-04

**Authors:** Jonathan Chevrier, Robert B. Gunier, Asa Bradman, Nina T. Holland, Antonia M. Calafat, Brenda Eskenazi, Kim G. Harley

**Affiliations:** 1 Center for Children’s Environmental Health Research, School of Public Health, University of California, Berkeley, Berkeley, California, USA; 2 Division for Laboratory Sciences, National Center for Environmental Health, Centers for Disease Control and Prevention, Atlanta, Georgia, USA

**Keywords:** bisphenol A, endocrine disruption, neonates, pregnancy, thyroid hormone

## Abstract

Background: Bisphenol A (BPA) is widely used in the manufacture of polycarbonate plastic bottles, food and beverage can linings, thermal receipts, and dental sealants. Animal and human studies suggest that BPA may disrupt thyroid function. Although thyroid hormones play a determinant role in human growth and brain development, no studies have investigated relations between BPA exposure and thyroid function in pregnant women or neonates.

Objective: Our goal was to evaluate whether exposure to BPA during pregnancy is related to thyroid hormone levels in pregnant women and neonates.

Methods: We measured BPA concentration in urine samples collected during the first and second half of pregnancy in 476 women participating in the CHAMACOS (Center for the Health Assessment of Mothers and Children of Salinas) study. We also measured free thyroxine (T_4_), total T_4_, and thyroid-stimulating hormone (TSH) in women during pregnancy, and TSH in neonates.

Results: Associations between the average of the two BPA measurements and maternal thyroid hormone levels were not statistically significant. Of the two BPA measurements, only the one taken closest in time to the TH measurement was significantly associated with a reduction in total T_4_ (β = –0.13 µg/dL per log_2_ unit; 95% CI: –0.25, 0.00). The average of the maternal BPA concentrations was associated with reduced TSH in boys (–9.9% per log_2_ unit; 95% CI: –15.9%, –3.5%) but not in girls. Among boys, the relation was stronger when BPA was measured in the third trimester of pregnancy and decreased with time between BPA and TH measurements.

Conclusion: Results suggest that exposure to BPA during pregnancy is related to reduced total T_4_ in pregnant women and decreased TSH in male neonates. Findings may have implications for fetal and neonatal development.

Bisphenol A (BPA) is widely used in the manufacture of polycarbonate plastic bottles, epoxy resins used in the inner lining of food and beverage cans, thermal receipts, medical equipment, tableware, and water supply pipes. Approximately 2.4 billion pounds of BPA were produced in the United States in 2007 [U.S. Environmental Protection Agency (EPA) 2010], and 95% of U.S. women of reproductive age (18–44 years) from the 2007–2008 National Health and Nutrition Examination Survey (NHANES) had detectable BPA levels in their urine [Centers for Disease Control and Prevention (CDC) 2011]. Unconjugated BPA has been detected in cord blood, placental tissue, and amniotic fluid, suggesting that the chemical can cross the placenta (Ikezuki et al. 2002; Schonfelder et al. 2002). Following a review of BPA’s potential to cause adverse reproductive and developmental effects, the National Toxicology Program (NTP) published a report in 2008 expressing “some concern” (the midpoint of a five-level scale) that current human exposure to BPA resulted in adverse effects on brain development and behavior in fetuses, infants, and children (Chapin et al. 2008).

Thyroid hormones (TH) play an essential role in pre- and postnatal growth and brain development in humans. Although severe maternal and neonatal thyroid insufficiency or overactivity has been known to alter cognition, behavior, and growth for more than a century (Dunn 1993), more recent evidence suggests that mild alterations in thyroid function may also influence these outcomes (Pop et al. 1999). Potential effects of BPA on cognition and behavior may thus be attributable in part to disruption of thyroid function.

Experimental evidence offers some support for this hypothesis. For instance, one *in vitro* study reported that BPA antagonized the ability of TH to affect oligodendrocyte differentiation (Seiwa et al. 2004). Moriyama et al. (2002) also found that BPA binds to the thyroid receptor (TR), antagonizes triiodothyronine (T_3_) binding to the TR, and inhibits TR-mediated gene expression *in vitro*. Furthermore, an animal study suggested that oral exposure to BPA results in a nonmonotonic transitory decrease in free, but not total, thyroxine (T_4_) in pregnant rats (Xu et al. 2007). Prenatal exposure to BPA, on the other hand, was related to a transitory dose-related elevation in total T_4_ among both male and female pups in one study (Zoeller et al. 2005) and in a nonmonotonic increase in free T_4_ (at postnatal day 7) followed by a decrease (at postnatal day 21) among male pups only in another study (Xu et al. 2007); a third study found no effect of prenatal BPA exposure on total T_4_ (Kobayashi et al. 2005). The studies by Zoeller et al. (2005) and Xu et al. (2007) found effects at the lowest doses administered (1 mg/kg body weight and 0.1 mg/L drinking water, respectively). Studies conducted in nonpregnant animals generally found no effect of BPA on thyroid hormone levels (Nieminen et al. 2002; Seidlova-Wuttke et al. 2005).

Four human studies have examined relations between exposure to BPA and thyroid function, yielding conflicting results. Meeker et al. (2010) found no association between serum free T_4_, total T_3_, and thyroid-stimulating hormone (TSH) and BPA concentrations in urine samples collected the same day in 167 men seeking treatment at an infertility clinic in Boston, Massachusetts. However, when BPA urinary concentrations in samples collected at multiple time points (1–3 measurements taken 3–75 days apart) were considered, their geometric means (GM) were associated with depressed TSH. In addition, BPA urinary concentrations were positively associated with free T_3_ among factory workers with high occupational exposure (Wang et al. 2012). Sugiura-Ogasawara et al. (2005), on the other hand, found no significant relation between hypothyroidism (generally diagnosed based on elevated TSH levels) and BPA urine concentration among Japanese women with a history of recurrent miscarriages (8 hypothyroidism cases and 37 controls), but the authors did not examine relations with low TSH. Finally, in a recently published study based on NHANES data, Meeker and Ferguson (2011) found a marginally significant (*p* = 0.08) inverse relation between urine BPA concentration and total T_4_ among 1,367 adults ≥ 20 years of age (–5.4% per ln unit; 95% CI: 0.01, –11.3) but not among 329 adolescents 12–19 years of age. The association among adults reached statistical significance (*p* = 0.049) when sampling weights were ignored.

Although TH is essential to normal fetal and neonatal brain development and growth, no human studies have investigated associations between exposure to BPA during pregnancy and maternal or neonatal thyroid hormone levels. Our objective was thus to determine whether maternal exposure to BPA during pregnancy, estimated based on BPA urinary concentration, was associated with maternal or neonatal TH levels in a Mexican-American population living in the Salinas Valley, California.

## Methods

*Participants.* We collected data as part of the Center for the Health Assessment of Mothers and Children of Salinas (CHAMACOS), a longitudinal birth cohort study of environmental exposures and health among pregnant women and children. Women were eligible to participate in this study if they spoke Spanish or English, were ≥ 18 years old, had completed < 20 weeks gestation, and qualified for California’s low-income health insurance program. We enrolled participants (*n* = 601) between October 1999 and October 2000. We excluded women from the present analyses if they had twins (*n* = 5), had a miscarriage (*n* = 20) or stillborn baby (*n* = 3), were lost to follow-up (*n* = 40), were taking medication that could affect TH levels (*n* = 1), or did not have a BPA urine measurement during pregnancy (*n* = 28). TH was not measured in 169 mothers due to insufficient serum volume, leaving a final sample of 335 for the maternal analyses. For the analyses of neonatal TSH, we excluded 2 neonatal deaths and 138 neonates for whom TSH measures (*n* = 114) or age at heel stick (*n* = 24) were missing from medical records. A total of 364 mother–child pairs were included in the neonatal analyses; 476 women were included in at least one analysis. We obtained written informed consent from all participants and all research was approved by the University of California (UC), Berkeley, Committee for the Protection of Human Subjects before commencement of the study. CDC relied on the UC Berkeley Committee.

*Interviews.* We interviewed participants at enrollment (~ 13 weeks gestation) and near the end of the second trimester (~ 26 weeks gestation) using structured questionnaires. We collected information on demographics including maternal age, race/ethnicity, education, country of birth, and time lived in the United States. We also collected information on smoking, alcohol consumption, and drug use during pregnancy. In addition, we obtained data on iodine intake during pregnancy using a modified version of the Spanish-language Block 98 Questionnaire (Block et al. 1990; Harley et al. 2005)

*Sample analysis.* Measurement of thyroid hormone. We collected maternal blood samples during the second interview and processed them immediately after collection. Samples were stored at –80°C at the UC Berkeley School of Public Health Biorepository until analysis. A pilot study showed that the number of freeze–thaw cycles was related to an increase in free T_4_ (data not shown). Samples were thus thawed only once, shipped refrigerated and analyzed for TH by Quest Diagnostics’ Nichols Institute (San Juan Capistrano, CA) within 48 hr. Free T_4_ was measured using direct equilibrium dialysis followed by radioimmunoassay (Nelson and Tomei 1988), which provides accurate measurements despite pregnancy-induced elevations in T_4_-bound proteins (Nelson et al. 1994). Total T_4_ and TSH were measured in maternal serum using solid-phase immunochemiluminometric assays (Bayer ADVIA Centaur system; Siemens Healthcare Diagnostics, Deerfield, IL). The limits of detection (LODs) were 0.1 ng/dL for free T_4_, 0.1 µg/dL for total T_4_, and 0.01 mIU/L for TSH.

Neonatal TSH is routinely measured by the California Department of Health Services Genetic Diseases Branch (Richmond, CA) as part of the state’s Newborn Screening Program. Blood spots were collected soon after birth [median = 21 hr; interquartile range (IQR) = 17–26 hr] by heel stick on filter paper and were analyzed using a solid-phase, time-resolved sandwich fluoroimmunoassay (AutoDELFIA; PerkinElmer, Wellesley, MA). The LOD was 2 mIU/L. Neonatal TSH and age (hours) at the time of heel stick were abstracted from medical records by a registered nurse.

Measurement of BPA. We collected spot urine samples from participants in sterile, polypropylene, BPA-free urine cups during the first (12.4 ± 3.8 weeks gestation) and second half (26.2 ± 2.2 weeks gestation) of pregnancy. Samples were stored at –80°C until shipment on dry ice to the CDC (Atlanta, GA) for analysis. The total urinary concentration of BPA (free and conjugated species) was measured using online solid-phase extraction coupled with isotope dilution–high performance liquid chromatography–negative ion–atmospheric pressure chemical ionization–tandem mass spectrometry (Ye et al. 2005). The LOD was 0.4 µg/L. Blank samples as well as low (~ 2.8 μg/L) and high (~ 10 μg/L) concentration materials were included in each run as quality control measures. Analysis of field blanks showed no contamination by BPA using this collection protocol. To account for urine dilution, we determined creatinine concentration using a commercially available diagnostic assay (Vitros CREA slides; Ortho Clinical Diagnostics, Raritan, NJ) for all specimens and measured specific gravity using a hand-held refractometer (National Instrument Company, Baltimore, MD) for 88.9% and 94.9% of samples included in the maternal and neonatal TH analyses, respectively.

Measurement of other environmental chemicals. Lead concentration was measured in maternal blood by the California Department of Public Health by graphite furnace atomic absorption spectrophotometry. Serum polychlorinated biphenyls (PCBs), hexachlorobenzene (HCB), and polybrominated diphenyl ether (PBDE) flame retardants were measured by the CDC using gas chromatography/isotope-dilution high-resolution mass spectrometry (Castorina et al. 2011; Sjödin et al. 2004). We collected samples for these analyses at the end of the second trimester of gestation (mean ± SD = 27 ± 3 weeks gestation).

Statistical analysis. We used analysis of variance (ANOVA) to compare BPA urinary concentrations across variable categories and Pearson’s correlations to evaluate bivariate associations between continuous variables. Because animal studies have suggested nonmonotonic dose responses between BPA and TH levels (Xu et al. 2007), we ran generalized additive models (GAM) with a 3-degrees-of-freedom cubic spline including covariates selected for the final models, to evaluate the shape of exposure–response curves in our study population. None of the tests for digression from linearity were significant (*p* < 0.15), suggesting that relations did not depart from linearity. We therefore included linear terms for BPA in multiple linear regression models. We expressed exposure as the average of the two (first and second half of pregnancy) BPA measurements. BPA urine concentrations were heavily right-skewed and thus were log_2_-transformed to reduce the influence of outliers. We log_10_-transformed TSH to normalize residuals; free and total T_4_ were expressed on the arithmetic scale. Regression coefficients thus represent mean (free and total T_4_) or percent (TSH) change in outcomes for each doubling in BPA concentration. In addition, we ran multiple logistic regressions, categorizing TH as normal versus above or below their respective reference ranges.

We considered the following variables that may influence TH levels as potential confounders (expressed as shown in Table 1 or in parentheses): maternal age (continuous), race/ethnicity, education, family income, country of birth, number of years spent in the United States, parity, gestational age at the time of blood collection (for maternal TH analyses only; weeks, continuous), iodine intake (continuous), and smoking, alcohol consumption, and illegal drug use during pregnancy. For neonatal TSH analyses, we also considered sex, delivery mode, and age at the time of heel stick (hours, continuous). TSH surges at birth and sharply declines during the first few days of life (Brown et al. 2005). Our previous work suggests that age at the time of TSH measurement is a strong confounder for associations with other environmental contaminants in the CHAMACOS population (Chevrier et al. 2007, 2011). We thus used GAMs and applied cubic splines to this variable to minimize residual confounding. Finally, based on results from prior studies in this population (Chevrier et al. 2007, 2008, 2010), we considered environmental exposures such as (log_10_-transformed) blood lead (micrograms per deciliter), total serum PCBs, HCB, and PBDE flame retardants (nanograms per gram lipids) for inclusion in models. We included covariates in final models if they were associated with both TH and BPA (*p* < 0.20) in bivariate analyses. For analyses of maternal TH, final models comprised mother’s age, education level, country of birth, poverty level, alcohol and drug use during pregnancy, iodine intake, and HCB and PCB serum concentrations. Final models of neonatal TSH included mother’s country of birth and child’s age at thyroid hormone measurement.

**Table 1:** Geometric mean (GSD) of average urinary BPA concentrations (μg/g creatinine) during pregnancy by demographic characteristics in CHAMACOS study participants.

Characteristic	Maternal TH analyses (n = 335)			Neonatal TSH analyses (n = 364)		
	n (%)	GM (GSD)	n (%)	GM (GSD)
Maternal characteristics	
Age (years)
18–24	158 (47)	1.3 (2.0)	186 (51)	1.3 (1.9)
25–29	109 (33)	1.3 (1.8)	105 (29)	1.3 (1.8)
30–34	44 (13)	1.1 (2.2)	47 (13)	1.2 (2.2)
35–45	24 (7)	1.5 (2.3)	26 (7)	1.8 (2.9)
Race/ethnicity								
Caucasian	7 (2)	1.6 (2.0)	4 (1)	1.0 (1.5)
Latino	321 (96)	1.3 (2.0)	351 (96)	1.3 (2.0)
Other	7 (2)	1.8 (1.7)	9 (3)	2.0 (1.7)
Education								
≤ 6th grade	141 (42)	1.2 (2.0)	153 (42)	1.3 (2.0)
7–12th grade	117 (35)	1.3 (2.1)	130 (36)	1.3 (2.2)
≥ High school	77 (23)	1.3 (1.9)	81 (22)	1.3 (1.8)
Family income								
≤ Poverty line	205 (61)	1.2 (2.0)	217 (60)	1.3 (2.1)
Poverty line to 200%	117 (35)	1.3 (2.0)	133 (37)	1.3 (2.0)
> Poverty line	13 (4)	1.7 (1.6)	14 (4)	1.8 (1.6)
Country of birth								
United States	41 (12)	1.4 (2.0)	51 (14)	1.5 (2.1)
Mexico	287 (86)	1.2 (2.0)	305 (84)	1.3 (2.0)
Other	7 (2)	1.6 (1.8)	8 (2)	1.5 (2.0)
Time in United States (years)								
≤ 1	84 (25)	1.2 (1.9)	89 (24)	1.1 (1.9)
2–5	99 (30)	1.2 (2.1)	96 (26)	1.2 (2.1)
6–10	77 (23)	1.3 (1.9)	87 (24)	1.3 (2.0)
≥ 11	41 (12)	1.4 (2.1)	50 (14)	1.6 (2.2)
Entire life	34 (10)	1.5 (2.1)	42 (12)	1.5 (2.1)*
Parity								
0	111 (33)	1.2 (1.9)	134 (37)	1.2 (1.9)
≥ 1	224 (67)	1.3 (2.0)	230 (63)	1.4 (2.1)
Smoking during pregnancy								
Yes	23 (7)	1.5 (2.0)	17 (5)	1.4 (2.1)
No	312 (93)	1.2 (2.0)	347 (95)	1.3 (2.0)
Alcohol during pregnancy								
Yes	4 (1)	1.1 (1.5)	3 (1)	1.6 (1.0)
No	331 (99)	1.3 (2.0)	361 (99)	1.3 (2.0)
Illegal drug use during pregnancy								
Yes	5 (2)	1.3 (1.3)	5 (1)	2.1 (1.7)
No	330 (98)	1.3 (2.0)	359 (99)	1.3 (2.0)
Prepregnancy body mass index (kg/m2)								
< 25	128 (40)	1.3 (2.0)	136 (38)	1.4 (2.1)
25–30	128 (40)	1.2 (1.9)	138 (39)	1.2 (1.8)
> 30	66 (21)	1.3 (2.3)	82 (23)	1.4 (2.2)
Infant characteristics								
Sex								
Male	188 (52)	1.3 (1.9)
Female	176 (48)	1.3 (2.1)
Birth weight (g)								
< 2,500	13 (4)	1.6 (1.6)
2,500–3,500	189 (52)	1.2 (1.9)
> 3,500	162 (45)	1.3 (2.2)
Gestational age at birth (weeks)								
< 37	21 (6)	1.6 (1.9)
37–42	343 (94)	1.3 (2.0)
> 42	0 (0)	— (—)
*p < 0.05 based on analysis of variance (ANOVA).								

BPA has a short half-life in humans (< 6 hr) (Volkel et al. 2002), and some experimental studies suggest that exposure to BPA may have a transitory effect on TH (Xu et al. 2007; Zoeller et al. 2005). Given these data, we hypothesized that potential relationships between BPA and TH may be stronger when the time between the two measurements was shorter. We tested this hypothesis by conducting additional analyses using the BPA measurement that was closest, and the one farthest, in time to the TH measurement. For the neonatal TSH analysis, we also conducted stratified analysis by examining exposure in each trimester of pregnancy. Finally, we considered interaction by iodine intake dichotomized using the U.S. Institute of Medicine recommended daily allowance of 220 μg/day during pregnancy as the cutoff (Otten et al. 2006) based on the hypothesis that women with low iodine intake may be more susceptible to TH disruption. For neonates, we also examined effect modification by sex based on results from experimental studies (Xu et al. 2007) and by age (dichotomized at 24 hr). We set statistical significance at *p* < 0.05 for main effects and *p* < 0.10 for effect modification.

For concentrations below the LOD, we used values generated by the instrument when available. Otherwise (e.g., when no signal was detected), we imputed values at random based on a log-normal probability distribution whose parameters were determined by maximum likelihood estimation (Lubin et al. 2004). This method has been shown to yield reasonable estimates when detection frequencies are > 70%. We had complete data on most covariates. We imputed values of missing covariates (≤ 5%) at random based on observed probability distributions.

We conducted sensitivity analysis to evaluate the robustness of our results. We first re-ran models after excluding outliers identified using the generalized extreme studentized deviate many-outlier procedure (Rosner 1983). We also determined whether the method that we chose to adjust for urine dilution (i.e., by dividing urinary BPA by creatinine concentration) affected our results by running models with unadjusted (with and without controlling for creatinine in models) and specific gravity–adjusted BPA concentrations. Finally, we applied inverse probability weights in an attempt to account for selection bias due to exclusion from final models (Hernan et al. 2004). We determined weights by multiple logistic regression whose independent variables were selected based on a deletion–substitution–addition algorithm (Sinisi and van der Laan 2004). Estimates from all of the above models were similar (data not shown). We present results using creatinine-adjusted BPA and including outliers in unweighted regression models.

## Results

*Participant characteristics.* Study participants were primarily young (80% < 30 years of age) Latinas (96%) born in Mexico (84–86%) who had immigrated to the United States within 10 years of enrollment (74–78%) (Table 1). Most women had low income (60–61% below the federal poverty line) and educational attainment (77–78% did not complete high school), and many were multiparous (63–67%). Few women smoked (5–7%), consumed ≥ 1 alcoholic drink per week (1%), or used illegal drugs (1–2%) during pregnancy. There were slightly more male (52%) than female infants. Only 4% of infants were born at low birth weight (< 2,500 g), and 6% were preterm (< 37 weeks gestation). As many as 8.2% of women had iodine intakes below the recommended daily allowance.

*Maternal BPA urine concentrations.* BPA was detected in 82% of samples. Median BPA concentrations were similar in the first and second half of pregnancy (Table 2) as well as for BPA measurements closest and farthest in time to thyroid hormone measurements (medians = 1.1–1.2 μg/g creatinine) [see Supplemental Material, Table S1 (http://dx.doi.org/10.1289/ehp.1205092)]. BPA concentrations measured in the first and second half of pregnancy were weakly but significantly correlated with Pearson’s and intraclass correlation coefficients of 0.16 each (*p* < 0.01). Women who had lived their entire life in the United States (GM = 1.5 µg/g creatinine) had higher BPA urine concentrations than those who lived in the United States ≤ 1 year (GM = 1.1–1.2 µg/g creatinine) (Table 1). Median BPA concentrations in our study population were lower than in women of reproductive age (18–44 years) in the 2007–2008 NHANES sample (median = 1.9 μg/g creatinine; IQR = 1.2–3.4) (CDC 2011).

**Table 2:** Urinary BPA concentrations (µg/g creatinine) during pregnancy in CHAMACOS study participants in samples included in analyses of maternal (*n* = 335) and neonatal (*n* = 364) serum TH levels.

Timing of measurement	n	Percentile					Range	GM (GSD)	Detection frequency (%)
25th	50th	75th
Maternal TH analyses
First half of pregnancy	290	0.6	1.1	1.8	< LOD–27	1.1 (2.3)	82
Second half of pregnancy	317	0.7	1.1	1.9	< LOD–37	1.2 (2.2)	81
Pregnancy average	335	0.8	1.2	1.9	< LOD–19	1.3 (2.0)	82
Neonatal TSH analyses
First half of pregnancy	309	0.7	1.1	1.9	< LOD–27	1.1 (2.3)	83
Second half of pregnancy	344	0.7	1.1	1.8	< LOD–37	1.2 (2.2)	82
Pregnancy average	364	0.8	1.2	1.9	< LOD–24	1.3 (2.0)	82
GSD, geometric standard deviation. LOD = 0.4 µg/L.														

*Maternal and neonatal TH levels.* Mean (± SD) serum concentrations of free and total T_4_ were 0.8 ± 0.2 ng/dL and 10.6 ± 1.6 μg/dL in maternal samples, respectively. The GM was 1.2 mIU/L (GSD = 1.7) for maternal TSH and 5.6 mIU/L (GSD = 1.8) for neonatal TSH. Based on trimester-specific laboratory reference ranges, four women had elevated free T_4_ (> 1.6 ng/dL) and 39 had low TSH (< 0.5 and < 0.8 mIU/L in second and third trimesters, respectively); none had high total T_4_ (> 17.8 and > 20.1 μg/dL in the second and third trimester, respectively). Seven women had low free T_4_ (< 0.5 ng/dL), 13 had low total T_4_ (< 8.0 μg/dL) and two had elevated TSH (> 4.6 and > 5.2 mIU/L in the second and third trimester, respectively). Seventeen had high TSH based on National Academy of Clinical Biochemistry guidelines (> 2.5 mIU/L) (Mandel et al. 2005). TSH was elevated (> 25 mIU/L) in one of the neonates.

*Association between maternal BPA urine concentrations and maternal and neonatal TH levels.* Maternal urinary BPA concentrations were not associated with maternal free T_4_ or TSH, but were negatively associated with maternal total T_4_ (Table 3). This association was not statistically significant using the average of the two BPA measurements (β = –0.13; 95% CI: –0.29, 0.02) or using the BPA measurements taken farthest in time (median = 95 days; IQR = 63–116) to total T_4_ serum measurements (β = –0.06; 95% CI: –0.20, 0.08), but was significant when the BPA measurements taken closest in time to total T_4_ measurements (median = 6 days; IQR = 0–15) were examined (β = –0.13; 95% CI: –0.25, 0.00). Average maternal urinary BPA was also related to increased odds of low total T_4_ [odds ratio (OR) = 1.6; 95% CI: 1.1, 2.3] and low TSH (OR = 1.5; 95% CI: 1.1, 2.0). Similar to results from linear regressions, associations were stronger when the BPA and TH measurements were taken closer together relative to when measurements were taken farther apart (data not shown).

**Table 3:** Associations between maternal BPA urinary concentrations during pregnancy and TH levels in women and their neonates participating in the CHAMACOS study.

Outcome	Exposure time	Unadjusted models			Adjusted models β (95% CI)
		n	β (95% CI)		
Maternal TH analyses								
Free T4	Closest measurement	332	0.00 (–0.02, 0.02)	0.00 (–0.02, 0.02)
Free T4	Farthest measurement	332	0.01 (–0.01, 0.03)	0.01 (–0.01, 0.03)
Free T4	Pregnancy average	332	0.00 (–0.02, 0.03)	0.00 (–0.02, 0.03)
Total T4	Closest measurement	335	–0.11 (–0.25, 0.02)	–0.13 (–0.25, 0.00)*
Total T4	Farthest measurement	335	–0.05 (–0.18, 0.09)	–0.06 (–0.20, 0.08)
Total T4	Pregnancy average	335	–0.12 (–0.28, 0.04)	–0.13 (–0.29, 0.02)
TSHa	Closest measurement	335	–2.9 (–7.5, 2.0)	–3.5 (–8.2, 1.5)
TSHa	Farthest measurement	335	0.3 (–4.7, 5.4)	0.1 (–4.9, 5.5)
TSHa	Pregnancy average	335	–2.5 (–8.2, 3.6)	–3.3 (–9.2, 2.9)
Neonatal TSH analyses								
TSHa	Closest measurement	364	–2.4 (–7.3, 2.7)	–2.0 (–6.1, 2.2)
TSHa	Farthest measurement	364	–1.2 (–7.4, 5.4)	–0.5 (–5.2, 3.3)
TSHa	Pregnancy average	364	–1.7 (–8.1, 5.2)	–1.8 (–4.2, 0.8)
Coefficients represent the mean change (free and total T4) or percent change (TSH) in thyroid hormone levels for each doubling in maternal BPA urinary concentrations. Maternal TH models were adjusted for mother’s age, education level, country of birth, poverty level, alcohol and drug use during pregnancy, iodine intake, and hexachlorobenzene and polychlorinated biphenyl serum concentrations. Neonatal TSH models were adjusted for mother’s country of birth and child’s age at TSH measurement. aPercent change in TSH serum concentration calculated using the following formula: (10β–1) × 100. *p < 0.05.								

**Figure 1:**
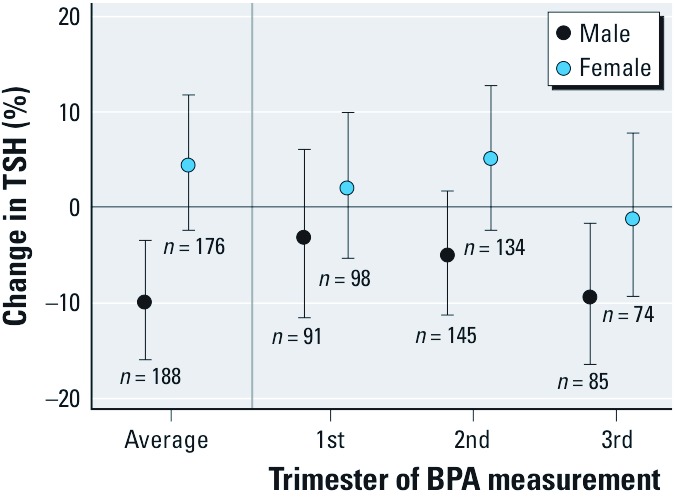
Change in neonatal TSH for each doubling in maternal urinary BPA concentration (µg/g creatinine) by infant sex and trimester of BPA measurement.

Although maternal BPA urinary concentrations were not associated with neonatal TSH when both sexes were combined (Table 3), effect modification by sex was statistically significant (*p* = 0.01). Analyses stratified by sex revealed an inverse relationship among boys (–9.9% for every doubling in average BPA; 95% CI: –15.9%, –3.5%) but not girls (4.4% for every doubling in average BPA; 95% CI: –2.4%, 11.7) (Figure 1). Among boys, associations with neonatal TSH were stronger when maternal BPA urinary concentrations were measured in the third trimester of gestation (–9.3% for every doubling in BPA; 95% CI: –16.3%, –1.7%) than when BPA was measured in the first (–3.2%; 95% CI: –11.5%, 6.0%) or second (–5.1%; 95% CI: –11.3%, 1.6%) trimesters. Associations were weaker as the time between the BPA and TSH measurements increased. Results were similar to those obtained using third trimester BPA when analyses were restricted to the BPA samples collected closest in time to the TSH measurements (median = 92 days; IQR = 81–104) (data not shown). None of the above associations were significantly modified by iodine intake or age at the time of heel stick (data not shown).

There were no statistically significant differences in demographic or lifestyle variables between participants included in maternal TH analyses relative to those excluded. Participants who were included in neonatal TSH analyses were more educated (22% ≥ high school education vs. 16%), were less likely to have used illegal drugs during pregnancy (2% vs. 5%), and delivered children with a higher birth weight (mean = 3,438 g vs. 3,314 g) (*p* < 0.05).

## Discussion

We report significant inverse associations between maternal BPA urine concentrations during pregnancy and TSH in male, but not female, neonates after adjustment for covariates. The relationship among males was stronger when BPA was measured in the third trimester of gestation. We also found an inverse association between maternal urinary BPA and serum total T_4_ when analyses were restricted to the BPA measurement taken closest in time to the TH measurement. However, contrary to a previous study investigating relations between urinary perchlorate and serum TSH and T_4_ using NHANES data (Blount et al. 2006), we found no evidence of a stronger relation among women with low iodine intake.

This is the first study to evaluate associations between maternal BPA urine concentrations during pregnancy and maternal or neonatal TH in humans. Meeker and Ferguson (2011) reported a borderline significant (*p* = 0.08) inverse association between total T_4_ in serum and BPA concentrations in the urine of 1,367 adult NHANES participants, consistent with our findings. However, no association was found with free T_4_ or total T_3_ (total T_4_ was not measured) in a smaller study of 167 men from an infertility clinic, but an inverse relation with TSH was reported (Meeker et al. 2010). On the other hand, BPA urine concentrations were not found to be related to hypothyroidism in 45 Japanese women with a history of miscarriage (Sugiura-Ogasawara et al. 2005). In addition to sample size, inconsistent results may be attributable to differences in study populations. Whereas one study was conducted in a nationally representative sample (Meeker and Ferguson 2011), other investigations examined the question in individuals who may have suffered from medical conditions (Meeker et al. 2010; Sugiura-Ogasawara et al. 2005). In addition, prior studies examined relations between BPA and TH among nonpregnant adults only. Fetuses and pregnant women may however be particularly susceptible to BPA exposure. Livers of pregnant rats have been shown to have a lower capacity to glucuronidate BPA relative to nonpregnant rats (Inoue et al. 2005), and the human fetus has limited glucuronidation capacity (Ring et al. 1999).

Of interest is the apparent sexually dimorphic relationship of BPA and neonatal TSH, suggesting an association in males but not females. Although data from NHANES suggested that inverse relations between BPA and total T_4_ were similar for both sexes in adults (Meeker and Ferguson 2011), one experimental rat study found that exposure to BPA during pregnancy and postpartum was related to alterations in free T_4_ (total T_4_ was not measured) among male pups only (Xu et al. 2007). Furthermore, results from some animal studies suggest that males may not metabolize BPA as efficiently as females. For instance, mRNA expression of the uridinediphosphate glucuronyl transferase (UDP-GT) isoform UGT 2B1 [which glucuronidates BPA in rats (Yokota et al. 1999)] has been reported to be lower in male rats than in females (Takeuchi et al. 2004). In addition, exposure to BPA was found to lower the expression of UGT 2B1 in male rats but not in females (Shibata et al. 2002).

BPA is eliminated quickly in humans (half-life < 6 hr) (Volkel et al. 2002), and data from our study as well as others’ (Braun et al. 2011; Ye et al. 2011), which show that the between-person variance of creatinine-adjusted BPA measurements accounted for only 9–16% of the total variance, suggest that a large number of measurements may be necessary to assess exposure over the long term. Urinary BPA measurements may, however, be adequate to evaluate transitory effects. Some experimental studies suggest that exposure to BPA may have such effects on TH (Xu et al. 2007; Zoeller et al. 2005). Given these results, we hypothesized that relations between BPA and TH may be stronger when the time elapsed between the two measurements was shorter. Our finding that the association between urinary BPA and maternal serum total T_4_ was significant when BPA was measured closer in time, but not when measurements were farther apart, and that the relation with neonatal TSH was stronger when BPA was measured in the third trimester of pregnancy appears to support this hypothesis or may be attributable to a specific developmental window of susceptibility to BPA. It is noteworthy that effect estimates for the average BPA and the closer measurements were very similar albeit of slightly different precision. Average urinary BPA may therefore remain a useful measure in studies of thyroid hormone disruption.

BPA may affect thyroid function through a number of pathways. In addition to binding and activating the TR (Moriyama et al. 2002), BPA has been shown to induce UDP-GT activity in European polecats (Nieminen et al. 2002). Since UDP-GT regulates the rate-limiting step in T_4_ metabolism in rats and presumably in humans, induction of this enzyme could underlie the reduction in T_4_ observed in this study. However, contrary to some hydroxylated metabolites of other commonly measured environmental chemicals, such as PCBs and PBDEs, BPA binds only weakly to the transport proteins transthyretin and thyroxine-binding globulin (Marchesini et al. 2008). The finding of a reduction in total T_4_ but not free T_4_ was unexpected. This may be attributable to a BPA-induced reduction in the serum concentration of transport proteins, an increased elimination of free T_4_ compensated by a release of T_4_ from transport proteins, or a hepatic sequestration of T_4_ as observed following exposure of mice to PCB-153 (Kato et al. 2011).

Strengths of this study include the availability of data on a large number of potential confounders including iodine intake during pregnancy. Iodine is an essential component of TH and both iodine deficiency and excess can adversely affect thyroid function (Delange et al. 2001). Data from NHANES suggest that, although the United States is generally considered an iodine sufficient area, a significant proportion of pregnant U.S. women are iodine deficient (Caldwell et al. 2008). We also conducted sensitivity analysis and our results were robust to methods used to adjust for urine dilution (creatinine or specific-gravity adjustment) and to adjustment for selection bias due to exclusion from final models.

This study also has some limitations. Associations that we report were identified in an immigrant Mexican-American population with low socioeconomic status. Our results thus need to be confirmed in other populations to evaluate their generalizability. Although we considered many variables known to affect TH levels, unmeasured confounders may have affected our results. In addition, the health implications of a possible decrease in maternal total T_4_ with no reduction in free T_4_ is unclear because bound T_4_ is not biologically active (Mendel 1989). Similarly, we are aware of no studies that investigated the developmental effect of lower but normal neonatal TSH.

In summary, we report an inverse relation between BPA concentration in maternal urine and maternal serum total T_4_ during pregnancy. Although we cannot rule out that average BPA concentrations during pregnancy may be relevant, the association of maternal BPA and total T_4_ was stronger when they were measured closer together relative to further apart in time, suggesting a transient effect of BPA. Similarly, the relationship of maternal BPA and neonatal TSH was strongest when maternal BPA was measured in the third trimester compared with earlier in pregnancy. This may also suggest a transient effect of maternal BPA on neonatal TSH, or alternatively, a developmental window of susceptibility. We recommend that future studies examine relations between prenatal exposure to BPA and TH in children and/or adults to elucidate this question.

## Supplemental Material

(115 KB) PDF
