# Supplementary material for: Maternal Urinary Bisphenol A during Pregnancy and Maternal and Neonatal Thyroid Function in the CHAMACOS Study
Source: Environ Health Perspect. 2012 Oct 4;121(1):138–44. doi: 10.1289/ehp.1205092 (PMC3553432; doi:10.1289/ehp.1205092)
Supplement: (115 KB) PDF [file ehp.1205092.s001.pdf]

SUPPLEMENTAL MATERIAL

Maternal Urinary Bisphenol A during Pregnancy and Maternal and Neonatal Thyroid Function in the CHAMACOS Study

Jonathan Chevrier[1], Robert B. Gunier[1], Asa Bradman[1], Nina T. Holland[1], Antonia M. Calafat[2], Brenda Eskenazi[1] and Kim G. Harley[1]

[1]Center for Children's Environmental Health Research, School of Public Health, University of California, Berkeley, Berkeley, California, USA.

[2]Division for Laboratory Sciences, National Center for Environmental Health, Centers for Disease Control and Prevention, Atlanta, Georgia, USA.

Supplemental Material, Table S1. Urinary bisphenol A concentrations (µg/g creatinine) during pregnancy in CHAMACOS study participants in samples included in analyses of maternal (n=335) and neonatal (n=364) serum thyroid hormone levels.

Timing of Measurement	N	Percentiles 25th	50th	75th	Range	Geometric Mean (GSD[a])	Detection Frequency
Maternal TH[b] analyses							
Closest measurement	335	0.7	1.1	1.9	<LOD[c] – 37	1.2 (2.3)	82%
Farthest measurement	335	0.7	1.1	1.8	<LOD – 27	1.1 (2.3)	81%
Pregnancy average	335	0.8	1.2	1.9	<LOD – 19	1.3 (2.0)	82%
Neonatal TSH[d] analyses							
Closest measurement	364	0.7	1.2	1.9	<LOD – 48	1.2 (2.3)	82%
Farthest measurement	364	0.7	1.1	1.8	<LOD – 27	1.1 (2.3)	82%
Pregnancy average	364	0.8	1.2	1.9	<LOD – 24	1.3 (2.0)	82%

[a] GSD = geometric standard deviation.
[b] Thyroid hormone.
[c] Limit of detection (LOD) = 0.4µg/L.
[d] Thyroid-stimulating hormone
